# Design and assessment of an adherence monitoring device for inhalers

**DOI:** 10.1186/2045-7022-3-S1-P9

**Published:** 2013-05-03

**Authors:** Richard Costello, Richard Reilly

**Affiliations:** 1 RCSI, Medicine, Ireland; 2 Trinity College, Dublin, Engineering, Ireland

## Background

Inhalers are widely used in the treatment of asthma. Non-adherence to inhalers is considered to be a contributing factor to poor control of this condition. Non-adherence with inhaled medications may arise because the drug is taken at incorrect times or when the correct steps in the use of the inhaler are not followed.

## Methods

We designed a device with a high fidelity microphone and small sized acoustic storage to make time stamped acoustic recordings of an individual’s use of an inhaler. We related the acoustic features to the various steps involved in the correct use of an inhaler. We established a minimum acoustic profile required to use the inhaler correctly, thereby objectively quantifying technique. A cohort of subjects with moderate/severe asthma (n=44) used a salmeterol/fluticasone discus inhaler with the device attached for a month. The subjects were instructed in the use of the inhaler.

## Results

There was almost a full correlation between the number of audio files in which drug priming occurred (n=1674) compared to the number of doses administered (n=1687). Analysis of the recordings indicated that 6 (18%) had missed more than 20% of doses and 7 (21%) had more than 20% of doses with an error. The most common error was that subjects blew into the device, after the drug was deployed and with sufficient force that the drug was dispersed (n=184). Overall, 19 (57%) had more than 20% doses with an error in timing or technique.

## Conclusion

These studies indicate that this device and associated processing may be useful for the management of conditions such as severe asthma.

